# Incidence of Adverse Psychiatric Events During Treatment of Inflammatory Bowel Disease With Biologic Therapies: A Systematic Review

**DOI:** 10.1093/crocol/otz053

**Published:** 2019-12-19

**Authors:** Avni Jain, Ruth Ann Marrie, Leigh Anne Shafer, Lesley A Graff, Scott B Patten, Renée El-Gabalawy, Jitender Sareen, James M Bolton, John D Fisk, Charles N Bernstein

**Affiliations:** 1 Department of Internal Medicine, Max Rady College of Medicine, Rady Faculty of Health Sciences, University of Manitoba, Winnipeg, MB, Canada; 2 Department of Community Health Sciences, Max Rady College of Medicine, Rady Faculty of Health Sciences, University of Manitoba, Winnipeg, MB, Canada; 3 Department of Clinical Health Psychology, Max Rady College of Medicine, Rady Faculty of Health Sciences, University of Manitoba, Winnipeg, MB, Canada; 4 Department of Community Health Sciences, Cumming School of Medicine, University of Calgary, Calgary, AB, Canada; 5 Department of Psychiatry, Max Rady College of Medicine, Rady Faculty of Health Sciences, University of Manitoba, Winnipeg, MB, Canada; 6 Department of Anesthesiology, Perioperative and Pain Medicine, Max Rady College of Medicine, Rady Faculty of Health Sciences, University of Manitoba, Winnipeg, MB, Canada; 7 Departments of Psychiatry, Psychology & Neuroscience, and Medicine, Dalhousie University, Halifax, NS, Canada

**Keywords:** adverse psychiatric events, meta-analysis, randomized controlled trials, biologic therapy

## Abstract

We conducted a systematic review and a fixed-effects meta-analysis to determine whether incident adverse psychiatric events (APE) including depression, anxiety, psychosis, or suicide were associated with biologic therapy in IBD. Six randomized controlled trials and a cohort study met criteria, reporting an incidence of APE in 4,882 patients. The risk difference per 100 person-months of any APE with a biologic medication was 0.01 (95% confidence interval = 0.00–0.02). There was insufficient evidence available in randomized controlled trials to conclude that biologic therapy in IBD is associated with an increased incidence of APE.

## INTRODUCTION

Inflammatory bowel disease (IBD) is a chronic illness that is usually diagnosed in early adulthood.^1^ Therefore, it is a disease process that affects patients for many years over the course of their lifetime. As with other chronic inflammatory diseases, there is a higher rate of co-morbid psychiatric illnesses such as depression and anxiety than in the general population.^2^ In the Manitoba IBD Cohort Study, persons with IBD were two times more likely to experience major depression (lifetime prevalence of 27%) compared with controls (12%).^3^ Elsewhere, the prevalence of depression has been estimated to be 20% in persons with IBD.^4^ More recently, we found an increase in psychiatric comorbidity for up to 5 years before diagnosis in those with IBD, when compared with matched population controls; these elevated rates persisted postdiagnosis.^5^ IBD has also been closely linked with anxiety, particularly when patients have flares of their disease.^6,7^ During periods of remission, anxiety rates have been estimated between 29% and 35%, but this can rise to as high as 80% during a flare.^8^ Psychiatric comorbidity has been associated with more severe IBD, with increased frequency of flares, and with poor treatment adherence.^9,10,11^

The use of biologic therapies in the management of IBD has been a mainstay of management for two decades, with increasing use in recent years.^12^ Other immunosuppressants, like corticosteroids, are still commonly used to treat IBD and have well-known adverse effects of mood disturbances.^13^ It has been proposed that the pathophysiology of these mood disturbances may be through the corticosteroid influence on the hypothalamic-pituitary-adrenal axis.^14^ The potential of adverse psychiatric events (APE) among those treated with biologic therapies, however, have received little study to date. It is unknown if the increased numbers of published research reports of psychiatric comorbidity in IBD reflect increased research into the association, a secular trend in psychiatric morbidity in the population in general, and hence, it would be expected to increase in IBD as well or secular changes in IBD management. Although the relationship between biologic therapy and psychiatric comorbidity has not been studied in IBD, a study exploring depression outcomes in persons with rheumatoid arthritis commonly being treated with an anti-TNF therapy found that responders to anti-TNF were less likely to have depression at 1 year follow up post-therapy initiation.^15^ Nonetheless, some responders and some nonresponders had depression at follow-up and there has been little investigation into whether some persons receiving anti-TNF may experience depression in relation to the therapy itself, rather than other disease processes.

Determining potential factors that increase an individual’s risk of developing depression, anxiety, psychosis, or suicidality can be pivotal in their care. Although it has been suggested that anti-TNF therapy, for instance, may improve cognitive-affective biases and reduce visceral sensitivity,^16^ there has been insufficient study about whether anti-TNF or other biologic therapy may trigger mental health disorders. An association has been suggested between an increased burden of disease and development of psychiatric morbidity,^17^ which is why the evaluation for association with treatment is pertinent. Although anti-TNF is thought to potentially reduce depression,^18^ TNF levels in depression are unpredictable and it is possible that paradoxical effects may be seen modulating TNF,^19^ as unexpectedly as was seen in heart failure studies.^20^ We conducted a systematic review and meta-analysis to assess the APE of the currently available biologic therapies used for IBD, including infliximab, adalimumab, golimumab, certolizumab, vedolizumab, and ustekinumab.

## METHODS

This systematic review was conducted using a protocol established in consultation with an information specialist and a gastroenterologist. The findings were reported using the PRISMA Harms checklist as guidance.^21^

### Populations, Interventions, Comparators, and Study Design

Studies were included if they met the following criteria: (1) randomized controlled trial (RCT) (for inclusion in the meta-analysis), and observational studies and case series if they had control groups; (2) published in English; (3) conducted in an adult population with a diagnosis of IBD receiving treatment with infliximab, adalimumab, golimumab, certolizumab, vedolizumab, or ustekinumab; (4) reported APE including depression, anxiety, suicide or suicidal ideation, or psychosis. These biologics were chosen based on those that were Health Canada approved for use for the treatment of IBD at the time of the study design.

### Outcome Measures

The primary outcome was the reporting of APE (depression, anxiety, suicide or suicidal ideation, or psychosis) during treatment with infliximab, adalimumab, golimumab, certolizumab, vedolizumab, or ustekinumab.

### Search Strategy

The search strategy was developed in consultation with a health information specialist and a gastroenterologist. A search of the databases MEDLINE, EMBASE, Cochrane Central Register of Controlled Trials, and Cochrane database of systematic reviews was conducted (Supplementary Table 1). Publications from the inception of the databases to August 2018 were included. Related terms were searched for in titles and abstracts of publications. MeSH and Emtree terms were are also searched for in MEDLINE and EMBASE, respectively. For information regarding unpublished adverse effects (particularly psychiatric adverse effects), completed trials were also searched for on Clinicaltrials.gov. All search results were imported into Mendeley and duplicates were identified and removed.

### Study Selection

Titles and abstracts were screened by two reviewers to determine whether they met the inclusion criteria. If there was disagreement about whether to include a study, then this was discussed by the reviewers until consensus was reached. A similar approach was used for review of the full text articles.

### Data Extraction

Data were extracted manually and entered into a standardized word document and verified by a second reviewer. The variables for data extraction were author, year, study design, treatment arms (biologic therapy administered), comparator, duration, eligibility criteria, participant characteristics, and the number of participants with APE and without APE (by APE type) that were enrolled in the study, the number that completed the study, and the method for ascertaining adverse events.

### Risk of Bias Assessment

The internal validity of the RCTs was assessed using the modified Cochrane collaboration risk of bias tool.^22^ This tool assesses six domains of the study including selection, allocation, detection, blinding, reporting, and attrition bias. The cohort study was assessed using the Newcastle-Ottawa Quality Assessment form.

### Analysis

To estimate the risk difference (RD) of APE among those using a biologic therapy for IBD compared with those not using a biologic therapy, we used the following data, extracted from each study: (1) number of people on therapy who had an APE, (2) number of people not on therapy (controls) who had an APE, (3) person-months among those on therapy, and (4) person-months among those not on therapy. In some cases, person-months was estimated from the information available. For example, where the study provided information on total duration of the study, number of people enrolled, and number of people who completed the study, but not the duration of follow-up for individuals who did not complete the study, we estimated that the person-months among those who did not complete the study was one-half of the total study duration. Risk was defined as the number of APE expected per time period and was estimated from the data as the number of people who had an APE, divided by the number of person-months in the respective study. Risk was calculated per 100 person-months of study observation. To be included in the pooled estimate of RD (per 100 person-months), the study required a control group. Using a fixed-effects meta-analysis, we estimated the pooled RD and 95% confidence interval of APE across all eligible studies. We present the results of the meta-analysis with a forest plot, showing the RD from each individual study, the overall pooled RD, and the weight that each study contributed to the pooled RD. Heterogeneity between studies was assessed by *I*^2^. A value of 0% indicates no heterogeneity between studies, that is, no variation in study results that cannot be explained by chance.^23^

Analyses were conducted using Stata 15.0 (College Station, TX *StataCorp* LP).

## RESULTS

### Study Selection

**FIGURE 1.**
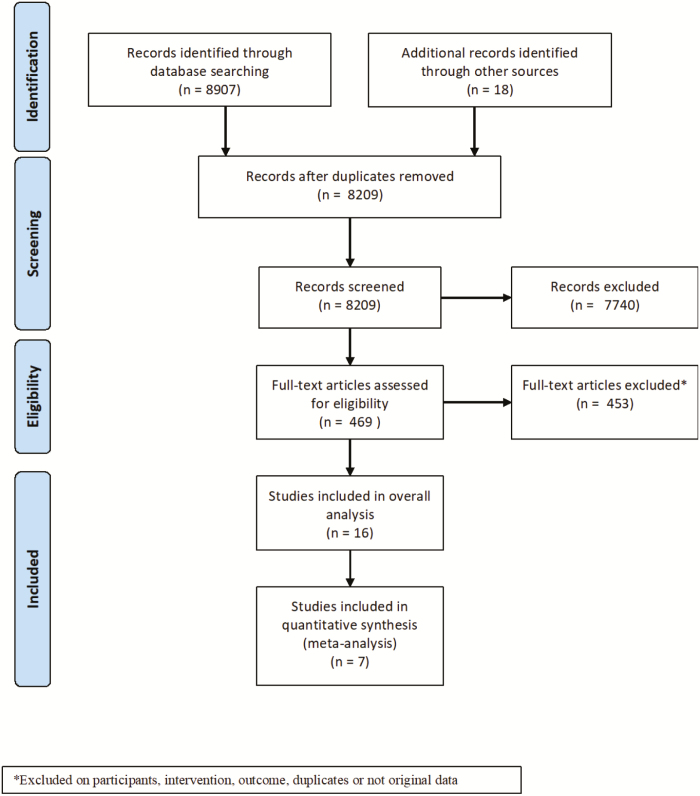
Flow diagram of study selection.

A total of 8,907 publications were identified through the database search and 18 through clinicaltrials.gov, of which 8,456 were excluded after titles and abstracts were screened. Of these, 865 were excluded based on intervention, 1,008 were excluded based on outcome, 2,782 were excluded based on study type, 1,764 were excluded based on study population, 1,319 were not original research, and 716 were duplicates (Figure 1). Two publications were not in English. Of 469 full-text articles that were subsequently assessed for eligibility, 16 met the inclusion criteria. Of these, 9 were open-label trials with no control group, so were excluded from meta-analysis of risk differences.

### Study Characteristics

The 16 studies included 15 clinical trials and 1 observational cohort study that were published between 2011 and 2018. Of the 15 clinical trials, 6 were RCTs and the remainder were non-randomized, open-label, uncontrolled trials (Supplementary Table 2). Most studies involved adalimumab^24,25,26,27,28^ (N = 5; 31.3%) and certolizumab^29,30,31,32^ (N = 4; 25.0%), followed by infliximab^33,34^ (N = 2; 12.5%), ustekinumab^35,36^ (N = 2; 12.5%), and vedolizumab^37,38^ (N = 2; 12.5%). Only one (6.3%) study involved golimumab.^39^ The six identified RCTs plus the one cohort study reported the incidence of APE in a total of 4,882 patients.

### Randomized Controlled Trials

In the six RCTs, there were 15 reported incidences of APE in 2,663 patients who were receiving the biologic therapy (0.49% of patients had an event).^24,28,35–37,39^ There were five reports of depression, six reported anxiety episodes, and four reports of suicidality. Psychosis was not reported nor mentioned at all in any of these studies.

### Adalimumab

There were two RCTs in which a total of 437 participants were exposed to adalimumab over the course of 52 weeks.^24,28^ One incident event of depression in the treated group was the only reported APE (0.23%).

### Certolizumab

No RCTs involving exposure to certolizumab reported APE.

### Ustekinumab

There was one RCT in which 131 participants were exposed to ustekinumab over 28 weeks.^35^ The most common APE was anxiety (N = 4; 3.10%), followed by depression (N = 2; 1.53%). In the other RCT involving ustekinumab,^36^ there were 394 participants exposed to ustekinumab over 8 weeks. The most frequent APE was suicide or suicide attempt (N = 2; 1.53%).

### Vedolizumab

There was one RCT in which 967 participants were exposed to vedolizumab over 52 weeks.^37^ The APE reported included depression (N = 2; 0.21%), anxiety (N = 2; 0.21%), and suicidality (N = 2; 0.21%).

### Golimumab

No RCTs involving exposure to golimumab reported APE.

### Infliximab

No RCTs involving exposure to infliximab reported APE.

### Observational Studies

#### Infliximab

There was one prospective, observational, cohort study in which 1,839 participants were exposed to infliximab over the course of 5 years.^34^ Depression was the most common APE (N = 10; 0.54%), followed by suicidality (N = 2; 0.12%) and anxiety (N = 1; 0.05%).

#### Open label, single group clinical trials

There were nine open label, single group clinical trials included.^25–27,29–33,38^ Of these, four involved certolizumab,^29–32^ three involved adalimumab,^25–27^ one involved infliximab,^33^ and one involved vedolizumab.^38^

The four studies with single group trials that involved certolizumab included a total of 987 participants.^29–32^ Depression was the most common APE reported at 0.51% (N = 5), followed by suicidality reported at 0.30% (N = 3), anxiety reported at 0.10% (N = 1), and psychosis reported at 0.10% of individuals (N = 1).

The three single group trials that involved adalimumab included a total of 2,000 patients, with study duration ranging from 20 to 288 weeks. The most commonly reported APE was depression at 0.2% (N = 4), followed by suicidality at 0.1% (N = 2) and psychosis at 0.05% (N = 1).^25–27^ There was one single group trial involving infliximab with 65 patients treated, 7.7% (N = 5) of whom reported depression as an adverse psychiatric event.^33^

There was also one single group trial involving vedolizumab with 72 patients, in which 2.8% (N = 2) reported depression.^38^

Of the studies that specifically described the method by which this primary outcome was determined, there were various descriptions of how APE were measured. D’Haens et al evaluated all adverse events through the treating physician utilizing a standardized form at every 6-monthly visit.^34^ Patients were asked to describe all adverse events that occurred since the previous visit. They recorded events for seven prespecified categories including serious infections, infusion-related reactions, hematological conditions, congestive heart failure, demyelinating neurological disorders, lymphoproliferative disorders and malignancies, fatalities, and other serious adverse events. Possibly, patients would have self-reported APE under the category of other serious adverse events; however, a standardized survey tool specifically for APE was not used. Sandborn et al had directed questionnaires to monitor for signs of progressive multifocal leukoencephalopathy; however, it is unclear how the remainder of the adverse events were measured.^37^ The remainder of the RCTs included in the meta-analysis did not explicitly state the method by which adverse events were measured, particularly APE. In regard to the open-label trials, Travis et al used a structured questionnaire to assess adverse events.^25^ The questionnaire included diagnosis or symptoms of the adverse event, onset date, duration and severity of the event, investigator opinion of the relationship of the adverse event to the biologic therapy, and the action taken. Sandborn et al stated that adverse events were assessed by the investigator through observation and questioning of the patients.^32^ Adverse events were then classified and descriptively summarized by primary system organ class and preferred terms according to the Medical Dictionary for Regulatory Activities. Hence, no study included in this systematic review used a validated tool for assessing an APE.

### Risk of Bias/Quality Assessment

#### Randomized controlled trials

Of the six RCTs included as part of the meta-analysis, one was assigned high risk of bias,^36^ two had a low risk of bias,^24,39^ and three were classified as having an unclear risk of bias (Supplementary Table 3).^28,35,37^ The absence of blinding was the major reason that one trial was assigned as having a high risk of bias.^36^ The unpublished trial was assigned to unclear risk of bias due to lack of available information.^28^ Two other trials were also assigned to unclear risk due to insufficient evidence about selective reporting.^35,37^

#### Cohort study

The one cohort study included in the meta-analysis^34^ was assessed using the Newcastle-Ottawa Quality Assessment Scale and was deemed to be good quality based on Agency for Healthcare Research and Quality (AHRQ) standards.

### Meta-Analysis of Risk Difference

**FIGURE 2.**
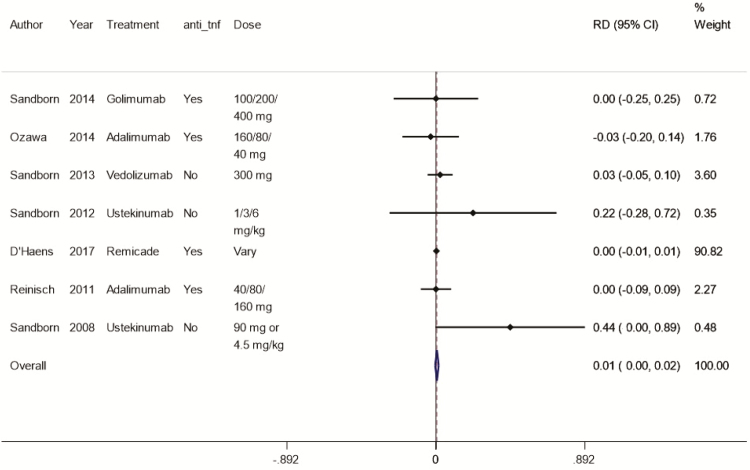
Risk difference (per 100 person-months) of any adverse mental health outcome, depending on therapy or not.

The pooled risk difference of any APE, comparing those with a biologic medication to those without, was 0.01 (95% CI: 0.00–0.02; Figure 2). In the cohort study involving infliximab,^34^ the patients who were exposed to the medication reported 1 anxiety event, 10 depression events, and 2 suicidality events. This cohort study had a substantially larger sample size than all other studies and thus contributed 90% of the weighted pooled result in meta-analysis. When we removed this study from analysis, the RD was 0.04 (95% CI: −0.03–0.10; Supplementary Figure 1).

The *I*^2^ value was 0% indicating no variation between studies that could not be explained by chance.

## DISCUSSION

The seemingly bidirectional relationship between inflammatory illness and psychiatric disease has become an increasingly important focus of research. A recent publication suggested that elevated levels of pro-inflammatory cytokines, such as interleukin-6 and TNF-alpha,^40^ may have an association with depression^41^ through their immunoregulating and modulating functions of the neural system. Another study showed that treatment of depressive symptoms through cognitive behavioral therapy reduced serum interleukin-6 levels in a small group of women,^42^ supporting the proposed association. This supports the importance of researching the effects of TNF and other cytokine modulating therapies and psychiatric events.

For individuals with IBD, psychiatric co-morbidity remains a management challenge and therefore it is essential to understand indicators of increased risk. We reviewed 16 studies involving infliximab, adalimumab, golimumab, certolizumab, vedolizumab, and ustekinumab for associations of these biologic therapies with APE. Depression and anxiety were comparably reported as APE although most occurred with an incidence of <1%, despite a high degree of variability in the incidence of APE across the included studies. The estimated risk difference per 100 person-months was 0.01, but the confidence interval crossed 0. Hence, there was insufficient evidence to conclude that biologic therapy use was associated with an increased risk of APE. This trend held true, with or without the inclusion of the D’Haens et al study,^32^ which had the greatest impact on the pooled results. Although our findings could be considered reassuring for physicians when they provide biologic therapies as part of their care of individuals with IBD, it is important to note that we also found a relative lack of reporting of APE in biologic therapy trials in IBD.

Although this was a comprehensive systematic review, only a limited number of studies were included in the meta-analysis. Most of the trials designed to study efficacy of biologic therapy in IBD did not report APE. It is unclear whether this was due to zero APE occurring, or whether a lack of adequate evaluation for this category of adverse outcome contributed to the inconsistencies in adverse event reporting among these large trials. Since some studies asked about adverse effects in open-ended ways, it is possible that patients may not consider elevated mental health symptoms as an adverse effect, especially when followed by a list of medical conditions. There were no specific descriptions of how the APE were assessed in each of these studies, but rather there were descriptions of general screening measures for any adverse events. There was also no indication in any of the studies as to whether the included participants were screened for psychiatric disease prior to initiation of the biologic therapy. Therefore, it is possible that the unknown presence of preexisting psychiatric disease confounded the results. This review has other limitations. The risk of bias assessment resulted in only two studies in the meta-analysis being of low risk. Many of the studies had unclear bias due to incomplete reporting of methods. By only including studies that were published in English, it is also possible that relevant trials published in other languages were missed. There may have also been unpublished data on regulatory websites other than clinicaltrials.gov.

No trials, to date, have been designed specifically to evaluate a possible association of APE with biologic therapies. Although our meta-analysis does not suggest an association between biologic therapy use and increased psychiatric disease incidence, this possibility cannot yet be excluded and longitudinal, observational studies are required to further evaluate this issue. Specifically, a study design that uses validated screening tools for APEs to evaluate the same patient through active disease, inactive disease, on and off of a biologic medication could be a potential direction of future research. Specifically, future studies should use validated screening tools for depression and anxiety, such as the Patient Health Questionnaire-9, or PROMIS tools, to evaluate the same patient through active disease, inactive disease, on and off of a biologic medication.^43^ Clinical trials should explicitly capture psychiatric symptoms on adverse event reporting forms.

In conclusion, our meta-analysis did not identify any association between biologic therapies used in the treatment of IBD and APE of depression, anxiety, psychosis, and suicidality. However, further research is required before this can be entirely excluded.

## Supplementary Material

otz053_suppl_Supplementary_Material

## Notes

Funding: This study was funded (in part) by the Canadian Institutes of Health Research (CIHR), Crohn’s and Colitis Canada, the Waugh Family Chair in Multiple Sclerosis (to R.A.M.), a Manitoba Research Chair from Research Manitoba (to R.A.M. and J.S.), and the Bingham Chair in Gastroenterology (to C.N.B.). Janet Rothney was the health information specialist who was consulted for developing the search strategy.

Conflicts of Interest: Charles Bernstein has served on advisory Boards for AbbVie Canada, Ferring Canada, Janssen Canada, Shire Canada, Takeda Canada, and Pfizer Canada; Consultant for Mylan Pharmaceuticals; Educational grants from Abbvie Canada, Pfizeer Canada, Shire Canada, Takeda Canada, and Janssen Canada; and Speaker’s panel for Abbvie Canada, Ferring Canada, Medtronic Canada, and Shire Canada. He has received research funding from Abbvie Canada. No other authors have potential conflicts of interest to declare.

Authors’ Contributions: Concept and design (A.J., R.A.M., L.A.S., and C.N.B.), data analysis (A.J., R.A.M., L.A.S., and C.N.B.), manuscript first draft (A.J.), and manuscript editing and revisions (A.J., R.A.M., L.A.S., L.A.G., S.B.P., R.E.G., J.S., J.M.B., J.D.F., and C.N.B.).
