# Supplementary material for: Incidence of Adverse Psychiatric Events During Treatment of Inflammatory Bowel Disease With Biologic Therapies: A Systematic Review
Source: Crohns Colitis 360. 2019 Dec 19;2(1):otz053. doi: 10.1093/crocol/otz053 (PMC6977711; doi:10.1093/crocol/otz053)
Supplement: otz053_suppl_Supplementary_Material [file otz053_suppl_supplementary_material.pdf]

Supplementary Figure 1. Risk Difference (per 100 person-months) of any Adverse Mental Health Outcome, Depending on Therapy or not (excluding D'Haens study)

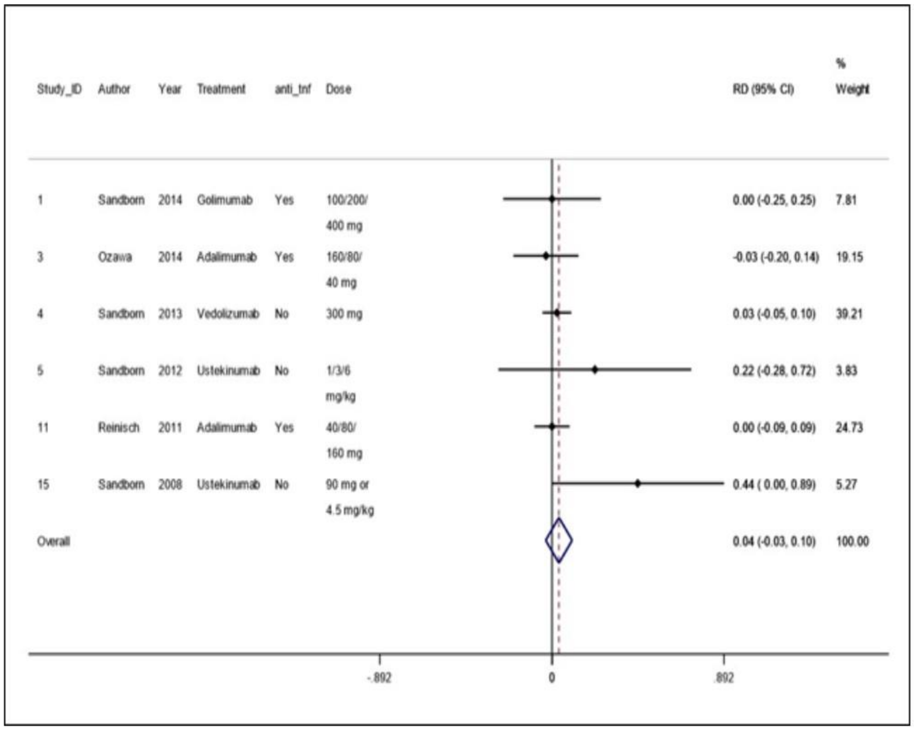

Supplement 1 APPENDIX 1

Database search

MEDLINE

1. Colitis, Ulcerative/
2. Crohn Disease/
3. crohn* disease.ti,ab.
4. ulcerative colitis.ti,ab.
5. Inflammatory Bowel Diseases/
6. inflammatory bowel disease.ti,ab.
7. Infliximab/
8. remicade.mp.
9. Adalimumab/
10. humira.mp.
11. golimumab.mp.
12. simponi.mp.
13. Certolizumab Pegol/
14. cimzia.mp.
15. Ustekinumab/
16. stelara.mp.
17. vedolizumab.mp.
18. entyvio.mp.
19. 1 or 2 or 3 or 4 or 5 or 6
20. 7 or 8 or 10 or 11 or 12 or 13 or 14 or 15 or 16 or 17 or 18
21. 19 and 20
22. limit 21 to (english language and humans)

EMBASE

Database search

1. ulcerative colitis/
2. Crohn disease/
3. inflammatory bowel disease/
4. infliximab/
5. remicade.mp.
6. adalimumab/
7. humira.mp.
8. golimumab/
9. simponi.mp.
10. certolizumab pegol/
11. cimzia.mp.
12. ustekinumab/
13. stelara.mp.
14. vedolizumab/
15. entyvio.mp.
16. 1 or 2 or 3
17. 4 or 5 or 6 or 7 or 8 or 9 or 10 or 11 or 12 or 13 or 14 or 15
18. 16 and 17

19. limit 18 to (human and english language)
20. 7 or 8 or 10 or 11 or 12 or 13 or 14 or 15 or 16 or 17 or 18

COCHRANE CENTRAL

1. MeSH descriptor: [Ulcerative colitis] explode all trees
2. MeSH descriptor: [Crohn disease] explode all trees
3. MeSH descriptor [Inflammatory bowel disease] explode all trees
4. MeSH descriptor [adalimumab] explode all trees
5. MeSH descriptor [infliximab] explode all trees
6. MeSH descriptor [certolizumab pegol] explode all trees
7. infliximab:ti,ab,kw
8. adalimumab:ti,ab,kw
9. golimumab:ti,ab,kw
10. certolizumab pegol:ti,ab,kw
11. ustekinumab:ti,ab,kw
12. vedolizumab:ti,ab,kw
13. #1 or #2 or #3
14. #4 or #5 or #6 or #7 or #8 or #9 or #10 or #11 or #12
15. #13 and #14 [in Cochrane Reviews and trials]

Supplementary Table 1

Author (year)	Study Design	Treatment arms	Comparator	Duration	Eligibility criteria	Participant characteristics	Psychiatric Adverse Events	Number started<br>Number completed
Ozawa 2014	Phase 3 Multicenter, Randomized, Double-blind, Placebo-controlled clinical trial	SC Adalimumab 80mg at week 0 and then 40mg at week 2 and 40mg every other week from week 4-52<br>Switch to rescue therapy at week 8 if inadequate response (N=87)<br><br>SC adalimumab 160mg at week 0 and then 80mg at week 2 and then 40mg every other week from 4-52 (N=90)	Placebo (N=96)	52 weeks	Inclusion:<br>• Aged >15<br>• Confirmed UC diagnosis for >90 days<br>• Active UC with Mayo Score of 6-12 and endoscopy score 2-3 despite concurrent therapy with stable dose of po steroids or 90 days of AZA or 6-MP<br>Exclusion:<br>• Disease limited to rectum<br>• Indeterminate colitis or Crohn's disease<br>• History of colectomy, ostomy, or planned surgery<br>• Previous exposure to biologic therapy<br>• History of TB or malignancy<br>• Pregnancy<br>Positive C. Diff, fulminant colitis or toxic megacolon	**SC adalimumab 80mg → 40mg**<br>• Mean age: 44.4<br>• N (%) female: 37 (42.5%)<br>• Mean Mayo Score: 8.5<br><br>**SC adalimumab 160mg → 80mg**<br>• Mean age: 42.5<br>• N (%) female: 29 (32.2%)<br>• Mean Mayo Score: 8.6<br><br>**Placebo group**<br>• Mean age: 41.3<br>• N (%) female: 26 (27.0%)<br>• Mean Mayo Score: 8.5<br><br>All enrolled from Japan	Depression:<br>**SC adalimumab 80mg → 40mg**<br>• N (%): 1 (1.15%)<br>**SC adalimumab 160mg → 80mg**<br>• N (%): 0 (0%)<br>**Placebo group**<br>N (%): 1 (1.04%)	Started = 274<br>Completed = 191

Sandborn 2013	Phase 3, Randomized, Placebo-Controlled, Blinded, Multicenter clinical trial	IV vedolizumab 300mg at week 0 and then week 2 – Induction Phase Double Blind (N=220)<br><br>IV vedolizumab 300mg at week 0 and then week 2 – Induction Phase Open Label (N=747)<br>__________________<br><br>IV vedolizumab every 6-8 weeks (for those with clinical response to induction phase) (N=814)<br><br>IV vedolizumab induction and then placebo (N-=153)	Placebo (N=148)	52 weeks	Inclusion:<br>• Aged 18-80<br>• Diagnosis of moderately to severely active CD, involving ileum/colon<br>• Treatment failure with immunomodulators, other anti-TNF agents, steroids<br>• May receive therapeutic dose of conventional therapies for IBD<br>Exclusion:<br>• Abdominal abscess<br>• Colectomy, ostomy, stenosis, short bowel syndrome<br>• Non-permitted IBD therapies within 60 days<br>• Chronic Hep B or C<br>Active or latent TB	**Induction double blind**<br>• Mean age: 36.3<br>• N (%) female: 115 (52.2%)<br>• Ethnicity: White – 182 (82.7%), Black – 3 (1.36%), Asian – 35 (15.9%), Other – 0<br>• Mean disease duration: 9.2 years<br>• Mean CDAI: 327.3<br><br>**Induction Open label:**<br>• Mean age: 35.6<br>• N (%) female: 401 (53.6%)<br>• Ethnicity: White – 689 (92.2%), Black – 17 (2.27%), Asian – 35 (4.68%), Other – 6 (0.08%)<br>• Mean disease duration: 9.2 years<br>• Mean CDAI: 322.2<br><br>**Placebo group**<br>• Mean age: 38.6<br>• N (%) female: 79 (53.3%)<br>• Ethnicity: White – 124 (83.8%), Black – 3 (0.02%), Asian – 19 (12.8%), Other – 2 (1.35%)<br>• Mean disease duration: 8.2 years<br>Mean CDAI: 324.6	(Analyzed in groups different to those presented in baseline characteristics)<br>Depression:<br>**IV vedolizumab every 6-8 weeks**<br>• N (%): 1 (0.12%)<br>**IV vedolizumab induction→placebo**<br>• N (%): 1 (0.12%)<br>**Placebo**<br>• N (%): 0<br><br>Anxiety:<br>**IV vedolizumab every 6-8 weeks**<br>• N (%): 1 (0.12%)<br>**IV vedolizumab induction→placebo**<br>• N (%): 1 (0.12%)<br>**Placebo**<br>• N (%): 0<br><br>Suicide or attempt:<br>**IV vedolizumab every 6-8 weeks**<br>• N (%): 1 (0.12%)<br>**IV vedolizumab induction→placebo**<br>• N (%): 1 (0.12%)<br>**Placebo**<br>N (%): 0	Started = 1116<br>Completed = 1010
Sandborn 2012	Phase 2b, Multicenter, Randomized, Double-blind, Placebo-controlled, Parallel Group Study	Ustekinumab – Induction Phase Week 0-8: 1mg/kg (N=130)<br><br>Ustekinumab – Induction Phase Week 0-8: 3mg/kg (N=133)<br><br>Ustekinumab – Induction Phase Week 0-8: 6mg/kg (N=131)	Placebo (N=132)	8 weeks	Inclusion:<br>• Aged ≥18<br>• Crohn's disease ≥ 3 months duration<br>• Must have received Remicade, Humira, or Cimzia at treatment dose and failed treatment<br>• CDAI 220-450<br>Exclusion:<br>• Any kind of bowel resection within 6 months<br>• Pregnancy/nursing/planning pregnancy<br>• Received Remicade, Humira, Cimzia within 8 weeks before study<br>• Complications of Crohn's disease present that would make it difficult to assess response to drug<br>• History of or ongoing chronic or recurrent infection	**Ustekinumab 1mg/kg**<br>• Mean age: 38.8<br>• N (%) female: 83 (63.3%)<br>**Ustekinumab 3mg/kg**<br>• Mean age: 38.2<br>• N (%) female: 75 (56.8%)<br>**Ustekinumab 6mg/kg**<br>• Mean age: 39.4<br>• N (%) female: 83 (63.3%)<br>**Placebo**<br>• Mean age: 39.5<br>• N (%) female: 68 (51.5%)	Suicide or attempt:<br>**Ustekinumab 1mg/kg**<br>• N (%): 1 (0.77%)<br>**Ustekinumab 3mg/kg**<br>• N (%): 0<br>**Ustekinumab 6mg/kg**<br>• N (%): 1 (0.76%)<br>**Placebo**<br>N (%): 0	Started = 526<br>Completed = 414

D'Haens 2017	Observational Prospective Cohort Study	Remicade for naïve patients – varying doses (N=1541)<br><br>Switched to Remicade (N=298)	Standard therapy (no biologic) (N=1121)	5 years	Inclusion:<br>• Aged ≥18<br>• Active/fistulizing Crohn's disease and:<br>- failed steroids or<br>- needed steroids for last 6 months or<br>- luminizing or fistulizing Crohn's disease qualifying for Remicade<br>• Give consent<br>Exclusion:<br>• Pregnant/nursing<br>• Prior treatment with anti-TNF<br>• Active or untreated latent TB or other infections<br>• Moderate-severe heart failure (class III-IV)<br>• Lymphoproliferative disorders/malignancies<br>Participating in any other clinical trials	**Remicade**<br>• Mean age: 36.1<br>• N (%) female: 935 (60.7%)<br>**Switched to Remicade**<br>• Not reported<br>**Standard therapy**<br>• Mean age: 37.5<br>• N (%) female: 677 (60.4%)	Anxiety:<br>**Remicade**<br>• N (%): 0<br>**Switched to Remicade**<br>• N (%): 1 (0.34%)<br>**Standard therapy**<br>• N (%): 0<br><br>Depression<br>**Remicade**<br>• N (%): 9 (0.39%)<br>**Switched to Remicade**<br>• N (%): 1 (0.34%)<br>**Standard therapy**<br>• N (%): 4 (0.36%)<br><br>Suicide or attempt<br>**Remicade**<br>• N (%): 1 (0.06%)<br>**Switched to Remicade**<br>• N (%): 1 (0.34%)<br>**Standard therapy**<br>• N (%): 2 (0.18%)	Started = 2662<br>Completed = 1774

Sandborn 2008	Phase 2a, Randomized, Multicenter, Double-blind clinical trial	Placebo SC weekly for 4 weeks and then ustekinumab 90mg SC weekly for 4 weeks (N=26)<br><br>Ustekinumab 90mg SC weekly for 4 weeks then placebo SC weekly for 4 weeks (N=25)<br><br>Placebo IV at week 0 then ustekinumab 4.5mg/kg IV at week 8 (N=26)<br><br>Ustekinumab 4.5mg/kg IV at week 0 then placebo IV at week 8 (N=27)<br><br>Ustekinumab 90mg SC weekly for 4 weeks (N=14)<br><br>Ustekinumab 4.5mg/kg IV weekly for 4 weeks (N=13)	(see previous)	28 weeks	Inclusion:<br>• Age ≥18 years<br>• Moderately to severely active CD/fistulizing disease for atleast 6 weeks<br>• CDAI 220-450<br>• Population 1 must have had active disease despite 5-ASA, Abx, steroids, and/or immunomodulators including anti-TNF agents<br>• Population 2 must have had active disease and failed to respond to infliximab at max approved dose<br>Exclusion:<br>• Local manifestations of CD such as strictures, abscesses, other complications that may require surgery<br>• Intra-abdominal surgery within 6 months prior to study<br>• Treatment with TPN within 6 weeks of study	**Placebo SC/Ustekinumab 90mg SC**<br>• Mean age: 36.7<br>• N (%) female: 11 (42.3%)<br><br>**Ustekinumab 90mg SC/Placebo SC**<br>• Mean age: 36.5<br>• N (%) female: 10 (40.0%)<br><br>**Placebo IV/Ustekinumab IV**<br>• Mean age: 43.5<br>• N (%) female: 14 (51.9%)<br><br>**Ustekinumab IV/Placebo IV**<br>• Mean age: 43.1<br>• N (%) female: 12 (46.2%)<br><br>**Ustekinumab 90mg SC**<br>• Mean age: 46.9<br>• N (%) female: 6 (42.9%)<br><br>**Ustekinumab 4.5mg/kg IV**<br>• Mean age: 42.7<br>• N (%) female: 8 (61.5%)	Anxiety:<br>**Placebo SC/Ustekinumab 90mg SC**<br>• N (%): 0 (0%)<br>**Ustekinumab 90mg SC/Placebo SC**<br>• N (%): 2 (8.0%)<br>**Placebo IV/Ustekinumab IV**<br>• N (%): 0 (0%)<br>**Ustekinumab IV/Placebo IV**<br>• N (%): 1 (3.7%)<br>**Ustekinumab 90mg SC**<br>• N (%): 0 (0%)<br>**Ustekinumab 4.5mg/kg IV**<br>• N (%): 1 (7.69%)<br><br>Depression:<br>**Placebo SC/Ustekinumab 90mg SC**<br>• N (%): 0 (0%)<br>**Ustekinumab 90mg SC/Placebo SC**<br>• N (%): 0 (0%)<br>**Placebo IV/Ustekinumab IV**<br>• N (%): 1 (5.7%)<br>**Ustekinumab IV/Placebo IV**<br>• N (%): 1 (3.7%)<br>**Ustekinumab 90mg SC**<br>• N (%): 0 (0%)<br>**Ustekinumab 4.5mg/kg IV**<br>• N (%): 0 (0%)	Started = 131<br>Completed = 97
Sandborn 2014	Phase 2/3 Multicenter, Randomized, Placebo-controlled, double-bind clinical trial	SC golimumab 100mg at week 0 and then 50mg at week 2 (N=72)<br><br>SC golimumab 200mg at week 0 and then 100mg at week 2 (N=331)<br><br>SC golimumab 400mg at week 0 and then 200mg at week 2 (N=331)	Placebo (N=331)	6 weeks	Inclusion:<br>• Aged >18<br>• UC confirmed by biopsy<br>• Moderately to severely active disease by endoscopy<br>• Currently treatment with, or history of failure to respond to 5-ASA, po steroids, 6-MP, AZA<br>• Steroid dependency<br>• Negative stool culture<br><br>Exclusion:<br>• Active TB<br>• Prior exposure to biologic anti-TNF agent<br>• Likely to require colectomy within 12 weeks<br>• UC limited to rectum or <20cm of colon<br>Stoma/fistula, history of extensive colon resection	**SC golimumab 100mg → 50mg**<br>• Mean age: 40.9<br>• N (%) female: 32 (44.4%)<br><br>**SC golimumab 200mg → 100mg**<br>• Mean age: 40<br>• N (%) female: 151 (45.6%)<br><br>**SC golimumab 400mg → 200mg**<br>• Mean age: 40.7<br>• N (%) female: 130 (39.2%)<br><br>**Placebo group**<br>• Mean age: 39<br>• N (%) female: 156 (47.1%)	ZERO	Enrolled = 1065<br>Completed = 1048

Reinisch 2011	Phase 3, Multi-center, Randomized, Double-blind, Placebo-controlled clinical trial	Adalimumab 80mg at week 0, then 40mg at week 2 and every other week (N=130)<br><br>Adalimumab 160mg at week 0, then 80mg at week 2, then 40mg every other week (N=130)	Placebo (N=130)	52 weeks	Inclusion:<br>• Age ≥18 years<br>• Diagnosis of UC for >90 days<br>• Diagnosis of active UC confirmed with biopsy<br>• Active UC, May Score 4-12 and endoscopy subscore 2-3 despite treatment with oral steroids (≥20mg/day) and/or AZA or 6-MP<br>• Consent and compliance<br>• Generally good health - determined by principal investigator<br>Exclusion:<br>• History of subtotal colectomy/ileostomy or planning surgery<br>• Pregnant or breastfeeding, or may become pregnant<br>• Previous anti-TNF therapy or any biologic therapy<br>• Received cyclosporine, tacrolimus, MMF within 30 days prior to study<br>Received IV steroids within 14 days of screening endoscopy	**Adalimumab 80mg/40mg SC**<br>• Mean age: 41.6<br>• N (%) female: 52 (40.0%)<br>**Adalimumab 160mg/80mg/40mg SC**<br>• Mean age: 38.2<br>• N (%) female: 47 (36.2%)<br><br>**Placebo**<br>• Mean age: 38.9<br>N (%) female: 48 (36.9%)	No relevant adverse psychiatric effects<br><br>ZERO	Started = 576<br>Completed = 382
Travis 2017	Phase 3 Open-label Multicenter, single group assignment, Clinical trial	SC Adalimumab 160mg at week 0 and then 80mg at week 2 and then 40mg every other weeks from week 4. D/c at week 8 if no response (N=463)	None	6 months	Inclusion:<br>• Aged 18-75<br>• Confirmed UC diagnosis for >90 days<br>• Failed conventional treatment<br>• Active UC with a Physicians Global Assessment score of 2 or 3, and SIBDQ ≤45 at baseline<br>• Concurrent therapy with po steroid, AZA or 6-MP If stable doses<br>Exclusion:<br>• IV steroids within 14 days<br>• Cyclosporine, tacrolimus, MMF within 30 days<br>• Fulminant colitis or toxic megacolon<br>• Prior exposure to adalimumab<br>• Anti-TNF within 56 days, or primary non-responder to anti-TNF previously<br>History of colectomy, ostomy, or planned surgery	• Mean age: 41.8<br>• N (%) female: 206 (44.7%)	Depression:<br>• N (%): 1 (0.22%)	Started = 463<br>Completed = 353

Colombel 2017	Phase 2, Non-randomized, Open-Label Clinical Trial	Vedolizumab 2mg/kg IV (Days 1, 15, 43, then q8 weeks) (N=37)<br><br>Vedolizumab 6mg/kg IV (Days 1, 15, 43, then q8 weeks) (N=35)	None	78 weeks	Inclusion:<br>• Age 18-75<br>• Confirmed and active UC or Crohn's disease<br>• CDAI score 220-450 (Crohn's) or Mayo Score of 2-7 (UC)<br>• Candidate for biologic therapy per guidelines<br>• Up-to-date on cancer screening<br>• No severe systemic disease<br>• Evidence of abscess<br>• Consent and agreeing to comply<br>Exclusion:<br>• Low lymphocyte count<br>• Hx of neurologic illness<br>• Active or recent serious infections<br>• Recent treatment with a biologic<br>• Impending surgery<br>Previous reaction to vedolizumab or anti-human antibody titres ≥1:125	**Vedolizumab 2mg/kg IV**<br>• Mean age: 42.0<br>• N (%) female: 21 (56.7%)<br>• Ethnicity: White – 37 (100%)<br>• IBD type: CD – 0 (0%), UC – 37 (100%)<br><br>**Vedolizumab 6mg/kg IV**<br>• Mean age: 42.1<br>• N (%) female: 22 (62.8%)<br>• Ethnicity: White – 34 (97.1%), American Indian or Alaskan - 1 (2.9%)<br>• IBD type: CD – 19 (54.3%), UC – 16 (47.2%)	Depression<br>**Vedolizumab 2mg/kg IV**<br>• N (%): 0 (0%)<br>**Vedolizumab 6mg/kg IV**<br>N (%): 2 (5.71%)	Started = 72<br>Completed = 15
Abbvie 2018	Phase 3, Multi-center, Open-Label, Single group Clinical Trial	Adalimumab 40mg SC every week/every other week (N=592)	None	388 weeks	Inclusion:<br>• Age >18<br>• Successfully enrolled and completed either M06-826 (NCT00385736) or M06-827 (NCT00408629)<br>• Diagnosis of UC for >90 days prior to study<br>• Diagnosis confirmed by colonoscopy with biopsy or flexible sigmoidoscopy with biopsy, exclusion of infection<br>• Active UC, Mayo score of 6-12 points despite treatment with at least one of the following:<br>1) Stable steroid dose for at least 2 weeks prior to study if >20mg/day, or for at least 40 days if <20mg/day AND/OR<br>2) 90-day consecutive course of AZA or 6-MP at treatment doses. Stable dose for at least 28 days prior to study<br>• Generally good health as determined by principal investigator<br>Exclusion:<br>• No response to weekly adalimumab therapy in M06-826 (NCT00385736) or M06-827 (NCT00408629)<br>Pregnant or breastfeeding	**Adalimumab 40mg SC**<br>• Mean age: 41.6<br>N (%) female: 214 (36.6%)	Psychosis:<br>• N (%): 1 (0.17%)<br><br>Suicide or attempt:<br>N (%): 1 (0.17%)	Started = 585<br>Completed = 255

Sandborn 2011	Phase 3b, Multinational, Open-label, Single Group Clinical Trial	Certolizumab Pegol 200mg SC at week 0, 2, 4, then q4weeks (N=402)	None	272 weeks	Inclusion:<br>• Age 18-75<br>• Diagnosis of CD<br>• CDAI 225-450<br>• Completed the C87085 [NCT00552058] study at week 6<br>• Provided consent<br>• Committed to comply with TB prophylaxis if applicable<br>Exclusion:<br>• Previous treatment with anti-TNF medications<br>• Subject is experiencing serious adverse event<br>• Serious intercurrent illness/infection<br>• Evidence of TB on CXR<br>• Pregnant or breastfeeding, or may become pregnant<br>Expected to receive live virus/bacterial vaccination within 3 months before/after trial	**Certolizumab Pegol 200mg SC**<br>• Mean age: 37.3<br>N (%) female: 221 (55.0%)	Depression:<br>• N (%): 3 (0.75%)<br><br>Psychosis:<br>• N (%): 1 (0.25%)<br><br>Suicide or attempt:<br>• N (%): 1 (0.25%)	Started = 402<br>Completed = 87
Pollack 2011	Phase 3, Multi-Center, Non-Randomized Open-Label Clinical Trial	Adalimumab 40mg SC every other week or every week (N=945)	None	20 weeks	Inclusion:<br>• Age 18-75<br>• Diagnosis or moderate to severe Crohn's by endoscopy or radiology evaluation for >4 months<br>• Inadequate response to conventional therapy<br>• Otherwise in good health with stable medical history<br>• Harvey Bradshaw Index ≥7<br>Exclusion:<br>• Surgical bowel resection within 6 months or planning resection at any time during trial<br>• Pregnant or breastfeeding, or may become pregnant<br>• Previous adalimumab treatment<br>• Previous exposure to natalizumab<br>On prednisone >40mg/day or budesonide >9mg/day	**Adalimumab 40mg SC**<br>• Mean age: 35.3<br>N (%) female: 568 (60.1%)	Depression:<br>• N (%): 3 (0.32%)<br><br>Suicide attempt:<br>• N (%): 1 (0.11%)	Started = 945<br>Completed = 785

Feagan 2013	Phase 3 Non-Randomized, Open Label, Single Group Clinical Trial	Certolizumab pegol 400mg SC q2weeks or q4weeks (N=229)	None	164 weeks	Inclusion:<br>• Age ≥18 years<br>• Diagnosis of CD<br>• Previous treatment failure to infliximab (intolerance/no response)<br>• Completed study C87042 [NCT00308581] (previously treated with infliximab)<br>Exclusion:<br>• Subjects from countries where certolizumab is authorized in Crohn's disease treatment<br>• Previous anti-TNF therapy or any biologic therapy<br>• Strictures, recent bowel resection, protocolectomy, total colectomy<br>• Current TPN<br>Pregnant or may become pregnant	**Certolizumab pegol 400mg SC**<br>• Mean age: 31.8<br>N (%) female: 146 (63.8%)	Anxiety:<br>• N (%): 1 (0.44%)<br>Depression:<br>• N (%): 1 (0.44%)	Started = 233<br>Completed = 71
Ogata 2009	Phase 2, Non-Randomized, Multi-center, Open Label, Single Group Clinical Trial	Certolizumab pegol placebo every other week for 3 doses, then 400mg SC on weeks 8, 10, 12, 16, 20, 24, 28, 32 (N=18)<br><br>Certolizumab pegol 200mg SC every other week for 3 doses, then 400mg SC on weeks 8, 10, 12, 16, 20, 24, 28, 32 (N=13)<br><br>Certolizumab pegol 400mg SC every other week for 3 doses, then 400mg SC on weeks 8, 10, 12, 16, 20, 24, 28, 32 (N=15)	None	34 weeks	Inclusion:<br>• Age 16-64 years<br>• CD diagnosis for at least 24 weeks prior to study<br>• CDAI 220-450<br>• CRP >1 mg/dL<br>• Subjects who consented and did not show clinical efficacy at week 6 of NCT00291668 study<br>Exclusion:<br>• Subjects who experienced aggravation of CD during the above study and required treatment change<br>• Stoma<br>• Allergy to anti-TNF antibody<br>• Have participated in a certolizumab clinical study<br>Pregnant, lactating, may become pregnant	**Certolizumab pegol 400mg SC/Placebo**<br>• Mean age: 30.4<br>• N (%) female: 4 (22.2%)<br><br>**Certolizumab pegol 400mg SC/200mg**<br>• Mean age: 37.5<br>• N (%) female: 4 (30.8%)<br><br>**Certolizumab pegol 400mg SC/400mg**<br>• Mean age: 29.9<br>• N (%) female: 3 (20.0%)<br><br>All patients enrolled from Japan	Suicide or attempt:<br>**Certolizumab pegol 400mg SC/Placebo**<br>• N (%): 0 (0%)<br>**Certolizumab pegol 400mg SC/200mg**<br>• N (%): 0 (0%)<br>**Certolizumab pegol 400mg SC/400mg**<br>N (%): 1 (6.67%)	Started = 46<br>Completed = 26

Sandborn 2010	Phase 3, Multicenter, Open Label, Single group clinical trial	Certolizumab pegol 400mg SC at week 0, 2, 4 and then q4weeks (N=310)	None	360 weeks	Inclusion:<br>• Age ≥18 years<br>• CD definitive diagnosis for at least 3 months<br>• Participation in either of the CDP870-031 [NCT00152490] or CDP870-032 [NCT00152425] clinical studies in which the subject completed the Week 2 assessment in CDP870-031 [NCT00152490] or the Week 6 randomization in CDP870-032 [NCT00152425] but whose Crohn's Disease was significantly worse as determined by the investigator and whose Clinical Disease Activity Index (CDAI) score at entry to this study is either (subjects may have received active or placebo treatment):<br>- CDAI score 70 points higher at week 6 than at baseline<br>- CDAI score higher than baseline with absolute score of at least 350<br>• Gave consent<br>Exclusion:<br>• Presence of fistula/abscess<br>• Structuring disease with symptoms or signs of non-inflammatory mechanical obstruction or bowel perforation in the last 3 months<br>• Short bowel syndrome<br>• Functional colostomy/ileostomy<br>Positive stool culture for enteric pathogens	**Certolizumab pegol 400mg SC**<br>• Mean age: 36.5<br>N (%) female: 179 (57.7%)	Depression:<br>**Certolizumab pegol 400mg SC**<br>• N (%): 1 (0.32%)<br><br>Suicide or attempt:<br>**Certolizumab pegol 400mg SC**<br>• N (%): 1 (0.32%)	Started = 310<br>Completed = 24

Supplementary Table 2

Risk of bias/quality assessment

Study	Sequence Generation	Allocation Concealment	Blinding	Incomplete Outcome Reporting	Selective Outcome Reporting	Overall Risk of Bias
**Adalimumab**						
Ozawa 2014	Unclear	Unclear	Low	Low	Unclear	Unclear
Reinisch 2011	Low	Unclear	Low	Low	Low	Low
**Infliximab**						
D'Haens 2017	Good Quality (AHRQ standards)					
**Ustekinumab**						
Sandborn 2008	Low	Unclear	Low	Low	Unclear	Unclear
Sandborn 2012	Low	Low	High	Low	Low	High
**Vedolizumab**						
Sandborn 2013	Low	Low	Low	Unclear	Unclear	Unclear
**Golimumab**						
Sandborn 2014	Low	Unclear	Low	Low	Low	Low
